# Tumor Immunosenescence Driven by Chronic Inflammation: Mechanisms, Microenvironment Remodeling and Therapeutic Strategies

**DOI:** 10.14336/AD.2025.0471

**Published:** 2026-05-09

**Authors:** Yiran Liu, Yang Yu, Yuanyuan Chen, Jing Zhuang, Changgang Sun

**Affiliations:** ^1^College of Traditional Chinese Medicine, Shandong Second Medical University, Weifang 261000, China.; ^2^State Key Laboratory of Quality Research in Chinese Medicine, and Faculty of Chinese Medicine, Macau University of Science and Technology, Macau SAR 999078, China.; ^3^Department of Oncology, Weifang Traditional Chinese Hospital, Weifang 261000, China.

**Keywords:** Chronic inflammation, Immunosenescence, Senescence-associated secretory phenotype, Tumor microenvironment, Combination therapy

## Abstract

The development of malignant tumors, as one of the most challenging public health issues today, is closely related to the interaction of chronic inflammation and immunosenescence. This review systematically analyzes the multidimensional mechanism of chronic inflammation driving immunosenescence. Chronic inflammation triggers immunosuppression and drives immunosenescence through interactions with inflammatory signals, the senescence-associated secretory phenotype (SASP), metabolic reprogramming, and microbiome. These processes remodel the tumor microenvironment via a multidimensional interaction network, which significantly weakens the anti-tumor immune response and accelerates tumor progression. This review proposes targeting strategies focusing on source intervention, SASP network blockade, and combination therapy optimization, as well as precise regulation with the help of novel technologies to break through the limitations of traditional immune checkpoint inhibition and provide new breakthroughs for overcoming tumor immune escape.

## 1. Introduction

Malignant tumors, as a core challenge in global public health, have shown a continuous upward trend in morbidity and mortality [1]. According to statistics, the number of new cancer cases worldwide has exceeded 20 million in 2022 [2]. The accelerated aging of the population makes the situation of cancer prevention and control more and more serious [3]. It is worth noting that chronic inflammation plays a decisive role in the development of about twenty percent of malignant tumors, and forms “inflammatory soil” for tumor evolution by inducing genomic instability, inhibiting apoptosis, activating pro-survival signaling pathways, and reconfiguring the immunosuppressive microenvironment [4, 5]. Molecular epidemiological studies of malignant tumors with typical inflammatory etiological features, such as hepatitis-associated hepatocellular carcinoma, Helicobacter pylori-associated gastric cancer, and colitis-associated colorectal cancer, have further corroborated the centrality of chronic inflammation as a tumor initiator and persistent drive [6, 7]. In addition, the persistent release of reactive oxygen species (ROS) and cytokine storms in the chronic inflammatory microenvironment induces epigenetic reprogramming and inhibits cellular processes through the p16^INK4a^/p53 pathway, which puts immunosenescence into motion [8].

The essence of immunosenescence as a key hub connecting chronic inflammation and tumor immune escape is the compensatory depletion of the immune system with age or chronic inflammatory stimuli. Notably, this depletion is not simply an age-related decline, but an active pathological process mediated by chronic inflammation through SASP. This process is characterized by multilevel dynamics. At the organ level, thymic atrophy leads to a decrease in naïve T-cell production, which, together with lymph node structural disorders, weakens the anti-tumor immune response [9]; at the cellular level, the loss of T-cell receptor (TCR) diversity, dysfunction of dendritic cell antigen presentation, and the expansion of myeloid-derived suppressor cells (MDSCs) form the triple whammy of immunosuppression [10]; at the molecular level, the persistent secretion of SASP factors such as IL-6, TNF-α, and MMPs and the aberrant upregulation of immune checkpoint molecules such as PD-1, CTLA-4, TIM-3 constitute the key driving elements of the vicious circle [7, 11]. Driven by inflammation, immunosenescence leads to a weakening of anti-tumor immunosurveillance while promoting the infiltration of immunosuppressive cells, which creates conditions for immune escape from the tumor [12].

Although the NF-κB/ROS/SASP axis is widely recognized as a core pathway for inflammation-driven immunosenescence, the paradoxical and spatio-temporal heterogeneity of its regulatory network remains to be resolved [13]. For example, TGF-β signaling exhibits dual regulatory roles of promoting senescence and exerting anti-inflammatory effects in the hepatocellular carcinoma microenvironment [14]. Certain inflammatory mediators such as interferon gamma (IFN-γ), while delaying T cell senescence by activating the JAK/STAT pathway, also promote SASP activation in macrophages [15]. More importantly, how differences in the inflammatory senescence gradient between the tumor core and the invasive front influence immune cell fate decisions have not been fully elucidated. Therefore, understanding the biology of immunosenescence and its role in the tumor microenvironment, and deeply resolving the dynamic interplay network between inflammation and immunosenescence are crucial for developing precision therapeutic strategies based on the stage-specificity of immunosenescence.

**Figure 1.**
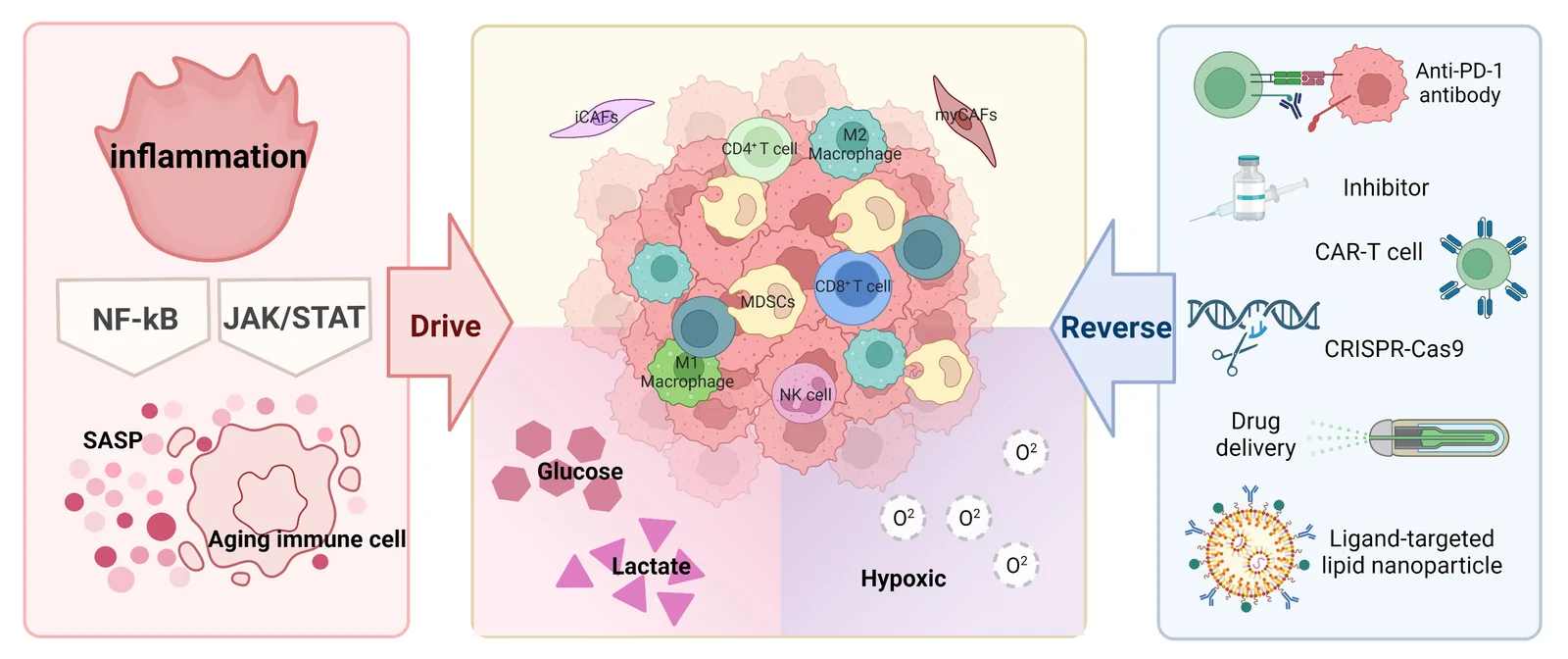
Molecular mechanisms and reversal strategies for inflammation-driven immunosenescence.

In this review, we will systematically elucidate the mechanisms by which chronic inflammation induces immunosenescence and promotes tumorigenesis and immune escape through multidimensional mechanisms, integrate the dynamic network of “inflammation-aging-immune escape”, and deeply analyze the dynamic remodeling of the tumor microenvironment (TME) in the context of immunosenescence. On this basis, we propose innovative therapeutic strategies based on spatial and temporal heterogeneity and targeting the “inflammation-aging” axis, aiming to provide a theoretical framework for deciphering the immune escape mechanism of tumors driven by chronic inflammation and immunosenescence, and laying the foundation for the development of new therapeutic modalities with both efficacy and safety (Fig. 1).

## 2. Molecular mechanisms of chronic inflammation-driven immunosenescence

### 2.1. Inflammatory signals

**Figure 2.**
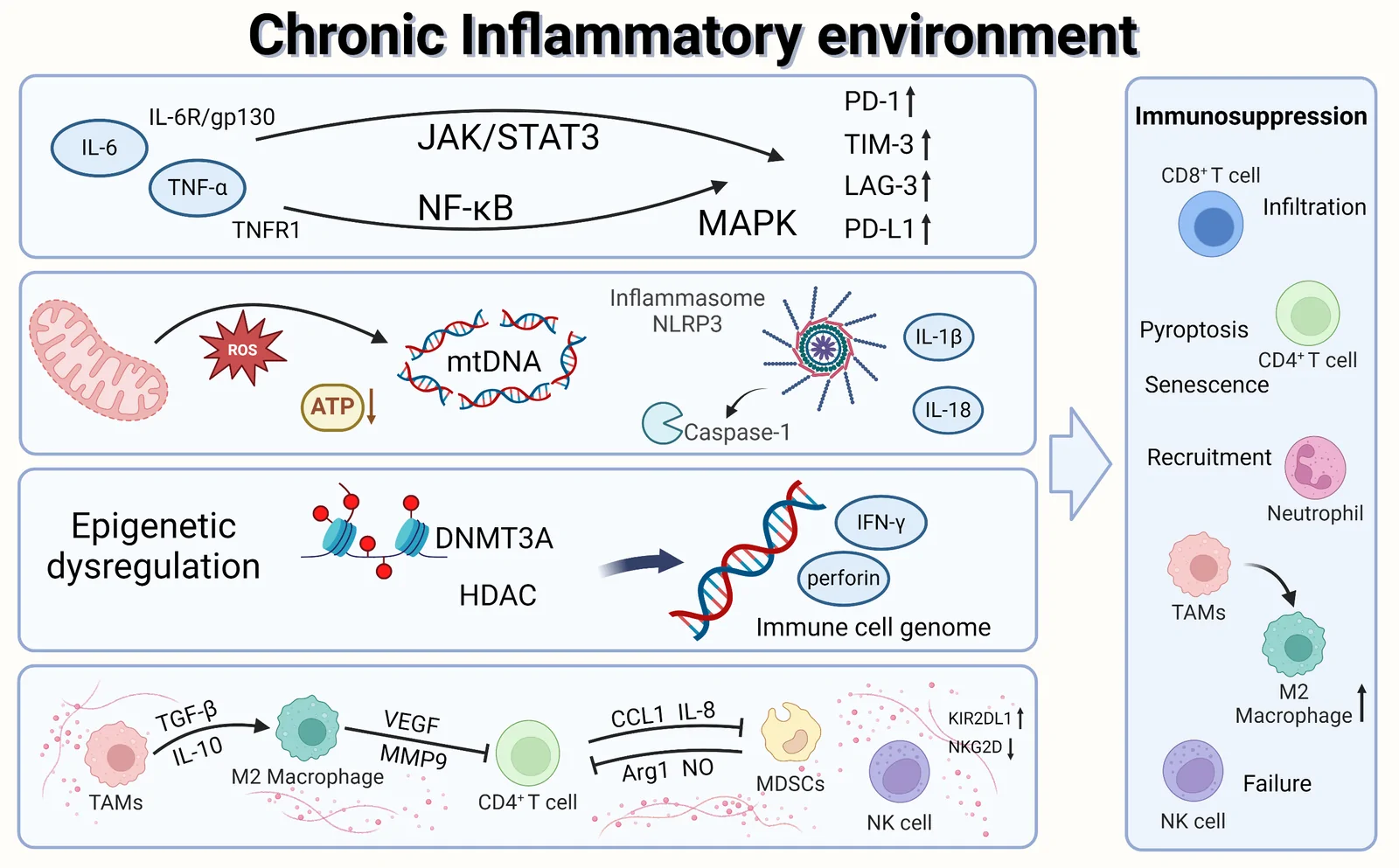
**Molecular network of chronic inflammation inducing immune cell functional depletion through multidimensional mechanisms.** Chronic inflammation activates pro-inflammatory signaling pathways, and the activation of these pathways, IL-6/JAK/STAT3 and TNF-α/NF-κB, leads to the up-regulation of immune checkpoint molecules, such as PD-1 and TIM-3, which in turn leads to T cell exhaustion. Meanwhile, oxidative stress is exacerbated, and mitochondrial dysfunction occurs in chronic inflammatory environments, and excessive ROS accumulation damages mtDNA, leading to metabolic failure. Not only that epigenetic regulation is also abnormal, for example, DNMT3A deletion results in hypermethylation of the IFN-γ gene and aberrant enrichment of the H3K27me3 modification, which drives T-cell differentiation toward the depleted phenotype. In addition, dynamic interactions between immune cells are involved, with CXCL1 secretion by immunosenescence T cells recruiting MDSCs and differentiation of TAMs to M2 phenotype inhibiting T cell infiltration.

Chronic inflammation induces functional exhaustion of immune cells through multidimensional mechanisms. Key contributors include aberrant activation of pro-inflammatory factors, oxidative stress, epigenetic dysregulation, and dynamic immune cell interactions (Fig. 2). These processes are interconnected to form a cascading pathological network that synergistically leads to the development of a T cell exhaustion phenotype, the loss of anti-tumor effector function, and the establishment of an immunosuppressive microenvironment, thereby weakening the body’s immune surveillance capability against tumors.

Cascading activation of pro-inflammatory signaling pathways is the initiating event of immune depletion. In chronic inflammatory environments, pro-inflammatory factors such as IL-6 and TNF-α directly induce functional depletion of immune cells by activating key signaling pathways [16]. IL-6 binding to the IL-6R/gp130 receptor complex activates the JAK/STAT3 pathway, which results in upregulation of the expression of immune checkpoint molecules, such as PD-1, TIM-3, and LAG-3, and the formation of a typical depletion phenotype (PD-1⁺ TIM-3⁺ LAG-3⁺). Meanwhile, the inhibition of IFN-γ and granzyme B secretion significantly impaired the anti-tumor activity of CD8⁺ T cells [17]. TNF-α activates the NF-κB signaling pathway through TNFR1, which on one hand induces the expression of apoptotic proteins such as BIM and FAS, and promotes T cell apoptosis; on the other hand, it upregulates the expression of PD-L1 on the surface of tumor cells, which results in the double-edged sword effect of immune escape [18, 19]. Notably, this immune depletion is not an isolated event, but rather a vicious cycle with subsequent SASP.

Oxidative stress and mitochondrial damage further exacerbate immunosenescence. Chronic inflammation-induced oxidative stress drives immunosenescence and functional decline of immune cells through excessive accumulation of ROS. ROS are mainly derived from the activation of NADPH oxidase NOX2 and leakage of the mitochondrial electron transport chain. Excess ROS oxidize mitochondrial DNA (mtDNA) and disrupts the mitochondrial membrane potential, leading to insufficient ATP synthesis and mitochondrial dysfunction, which in turn triggers T cell metabolic failure [20, 21]. Pilipow found that antioxidant metabolism modulates the stem cell properties and anti-tumor immune function of CD8⁺ T cells [22]. In addition, ROS promote IL-1β and IL-18 maturation in a caspase-1-dependent manner through the activated NLRP3 inflammasome, which induces T cell pyroptosis while recruiting neutrophils to form an inflammatory necrotic microenvironment [23]. In vitro and survival models demonstrated that ROS activate the NLRP3 inflammasome, leading to caspase-1-dependent maturation of IL-1β and IL-18. This process induces T cell pyroptosis and recruits neutrophils to establish an inflammatory necrotic niche [24]. However, low concentrations of ROS may maintain cellular homeostasis by activating antioxidant pathways, while high concentrations trigger irreversible damage. Precise regulation of ROS levels to balance immune activation and immunosenescence is a key direction for future research.

Abnormal epigenetic regulation is one of the central mechanisms of immune depletion. Deletion of the DNA methyltransferase DNMT3A leads to hypermethylation of the promoter regions of the IFN-γ and perforin genes in CD8⁺ T cells, silencing their transcriptional activity; it also prompts aberrant demethylation of depletion-associated genes, such as TOX and PDCD1, and promotes the differentiation of T cells toward a depleted phenotype [25]. Histone modification abnormalities are equally critical. Elevated histone deacetylase (HDAC) activity inhibits chromatin openness at the Foxp3 gene locus and hinders regulatory T cell (Treg) differentiation, whereas aberrant enrichment of the H3K27me3 modification promotes Th17 cell polarization and exacerbates the inflammatory vicious cycle [26, 27]. In addition, long noncoding RNAs, such as NEAT1, inhibit CD8⁺ T cell effector function by binding to EZH2, which provides a new target for epigenetic therapy [28]. Interestingly, recent single-cell ATAC-seq studies have revealed dynamic chromatin plasticity during T cell exhaustion. Epigenetic modifications remain reversible in the early stage of depletion, while they stabilize in the late stage [29]. This finding provides a theoretical basis for early intervention based on epigenetic reprogramming.

Dynamic interactions between immune cells play a central role in inflammation-driven immunosenescence. Senescent CD8⁺ T cells recruit MDSCs to the tumor site in a CXCR2-dependent manner through the secretion of CXCL1 and IL-8, which inhibit T-cell function in a localized immunosuppressive effects via arginase-1 (Arg1) and nitric oxide (NO) [30, 31]. Meanwhile, tumor-associated macrophages (TAMs) differentiate into M2-type under IL-10 and TGF-β stimulation, and secrete VEGF and MMP9 to promote angiogenesis and stromal remodeling, which further limit T cell infiltration [32, 33]. NK cell failure is another key component. Chronic inflammation induces downregulation of the NKG2D receptor, which impairs tumor recognition, and upregulation of the inhibitory receptor KIR2DL1, resulting in enhanced suppression of cytotoxic activity and impaired immune surveillance [34]. However, the spatio-temporal expression heterogeneity of inflammatory factors has not been sufficiently investigated, especially in different regions of the tumor, and how pro-inflammatory signals dynamically regulate immune depletion still needs to be deeply resolved.

### 2.2. SASP

The end state of immunosenescence is often accompanied by SASP activation, a process driven by a multilevel regulatory network that amplifies local inflammatory signals into a systemic pathological response, serving as a key hub connecting inflammation and immune-senescence. Senescent cells construct autocrine and paracrine signaling networks through the secretion of heterogeneous factors such as IL-6, TNF-α, and MMPs, which convert local inflammatory signals into systemic pro-tumorigenic effects and drive the remodeling of the TME [35]. In chronic inflammation-driven tumor ecology, immunosuppressive ecology is formed by senescent immune cells such as PD-1^+^ TIM-3^+^ LAG-3^+^ T cells, M2-type macrophages, and stromal cells such as cancer-associated fibroblasts (CAFs), which are further recruited to MDSCs through SASP factors and inhibit dendritic cell antigen-presenting function [36].

**Figure 3.**
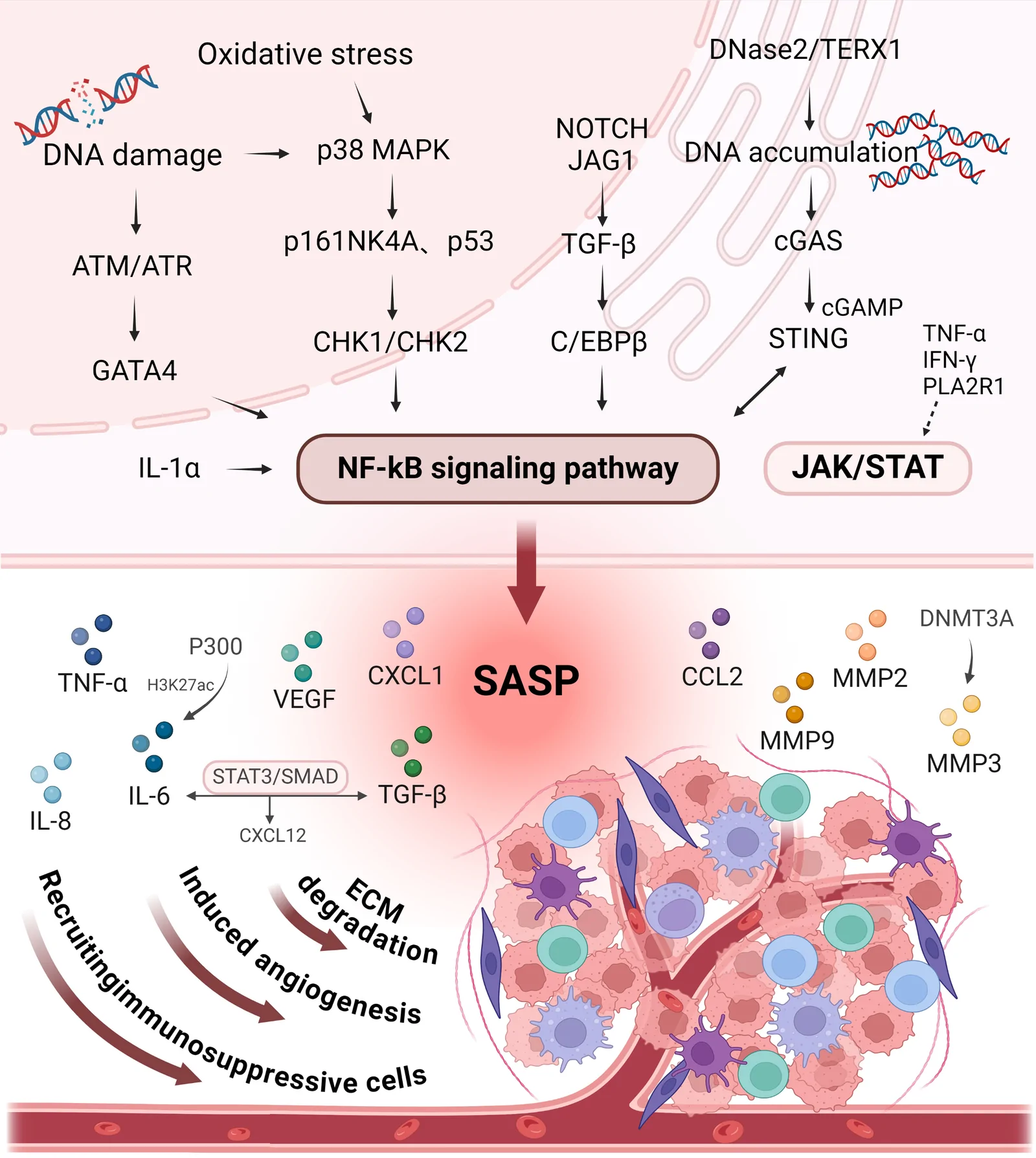
**Multi-level regulatory network of SASP factors and pro-tumorigenic effects.** The regulatory network of SASP is complex, on the one hand, the NF-κB and p38 MAPK pathways synergistically regulate the secretion of IL-6, IL-8 and other factors. On the other hand, ATM/ATR, cGAS-STING and NOTCH signaling pathways synergistically enhance the transcription of pro-inflammatory factors. In addition, epigenetic reprogramming is also involved, such as p300-mediated acetylation of H3K27, which amplifies the effects of SASP, which creates a vicious cycle of inflammation-aging-promotion through various mechanisms, such as recruitment of MDSCs, inhibition of dendritic cell antigen presentation, promotion of angiogenesis and metabolic inhibition.

The SASP regulatory core integrates NF-κB and p38 MAPK pathways (Fig. 3): NF-κB directly activates IL-6/IL-8 transcription, while p38 MAPK enhances SASP expression through C/EBPβ phosphorylation [37-39]. This network undergoes multilayered modulation — DNA damage responses sustain NF-κB via IKK complex activation, cytoplasmic dsDNA engages cGAS-STING signaling to boost inflammatory output, and IL-6/JAK/STAT3 feedback loops epigenetically silence NF-κB inhibitors [40-42]. Epigenetic mechanisms further exacerbate pro-inflammatory effects. In addition, mtDNA released from mitochondrial dysfunction triggers a type I interferon response via the cGAS-STING pathway, creating a positive feedback loop of inflammatory signaling [20, 43]. In pancreatic cancer, IL-6 and TGF-β secreted by tumor cells induce activation of CAFs and secretion of CXCL12 through the STAT3/SMAD pathway, which enhances the self-renewal capacity of cancer stem cells (CSCs). Spatial dynamics significantly influence SASP effects, as demonstrated by pancreatic cancer models where the differences in CXCL12 concentration dictate MDSC distribution and therapeutic responses [44].

SASP synergistically drives tumor progression through a multidimensional mechanism, forming a vicious cycle of “inflammation-aging-tumor promotion”. Table 1 provides the functional differences of the SASP factors in different tumor types. First, SASP recruits immunosuppressive cells. In pancreatic cancer, CXCL1/IL-8 recruits MDSCs through the CXCR2 axis, and its infiltration density positively correlates with the degree of tumor mesenchymal fibrosis and suppresses T cell function through arginase-1 [60]. Second, IL-6 and TGF-β synergistically downregulate the expression of the dendritic cell co-stimulatory molecule CD80/86, which impairs tumor antigen presentation and leads to the absence of tumor-specific T cell responses [61]. Matrix degradation programs and angiogenic factors collectively establish pro-metastatic niches, with clinical correlations observed in hepatocellular carcinoma where MMP overexpression predicts vascular invasion [62, 63].

Although the core regulatory network of the SASP (senescence-associated secretory phenotype) has been preliminarily elucidated, its functional plasticity remains a significant challenge. SASP factors exhibit context-dependent effects. IL-6, as a key SASP component, demonstrates dual roles: It can suppress tumors by activating immune cells, yet conversely promote tumor progression through pro-inflammatory and immunosuppressive mechanisms [64]. For instance, IL-6 displays pro-tumorigenic or anti-tumorigenic functions in breast and lung cancers [65, 66]. This duality complicates therapeutic strategies. Targeting IL-6 may inhibit its tumor-promoting effects in specific contexts but risks impairing its immune-activating functions, thereby reducing treatment efficacy [46]. Ultimately, IL-6’s effects depend on cell type, microenvironmental conditions, and concentration, making therapeutic outcomes unpredictable. Future studies must unravel its context-specific mechanisms to refine therapeutic approaches.

**Table 1:** Functional differences of SASP factors in different tumor types.

SASP factor	Tumor type	Functional differences	Reference
**IL-6**	Breast Cancer	Promotes tumor growth; correlates with dynamic homeostasis of CSCs; induces epithelial mesenchymal transition	[45]
	Liver cancer	Inhibits hepatocyte apoptosis and promotes tumor proliferation; induces endothelial cell proliferation and promotes tumor angiogenesis	[46]
	Prostate cancer	Promotion of EMT, invasion and metastasis through IL-6R/STAT3/miR-34a feedback loop	[17]
	Lung cancer	Promotion of PD-1 upregulation in T cells through the Rab37/IL-6/STAT3 transcriptional axis	[47]
**IL-8**	Breast Cancer	Induction of EMT; enhancement of CSCs activity	[45]
**TNF-α**	Breast Cancer	Synergizing with the NF-κB signaling axis to promote breast cancer cell survival and tumorigenicity; regulating metabolic reprogramming in breast cancer cells	[48]
**CXCL1**	Colorectal cancer	Promotes tumor cell proliferation, migration, and angiogenesis by stimulating the NF-κB/P300 signaling pathway	[49]
	Liver cancer	Promoting clonal growth of hepatocellular carcinoma cells and xenograft tumor formation through paracrine effects	[50]
**CCL2**	Liver cancer	Recruitment of CCR2(+) immature bone marrow cells for immunosurveillance of senescent hepatocytes	[51]
	Prostate cancer	Promotion of tumor cell growth survival and bone metastasis; CCL2 single nucleotide polymorphisms are associated with disease risk and aggressiveness	[52, 53]
**MMP2/MMP9**	Multiple tumors	Degrades extracellular matrix, disrupting the tumor invasion barrier and promoting metastasis and invasion; correlates with angiogenesis and supports tumor microenvironment remodeling	[54-56]
**VEGF**	Gastric cancer	VEGF expression increases with gastric cancer progression and correlates with the depth of tumor infiltration, which may be useful for early gastric cancer diagnosis	[57]
	Colorectal cancer	High VEGF expression promotes tumor recurrence and migration and affects cancer cell survival and multiplication through AKT-mediated signaling pathway	[58]
**TGF-β**	Multiple tumors	Inhibits immune cell activity and promotes immune escape	[59]

### 2.3. Metabolic reprogramming

**Figure 4.**
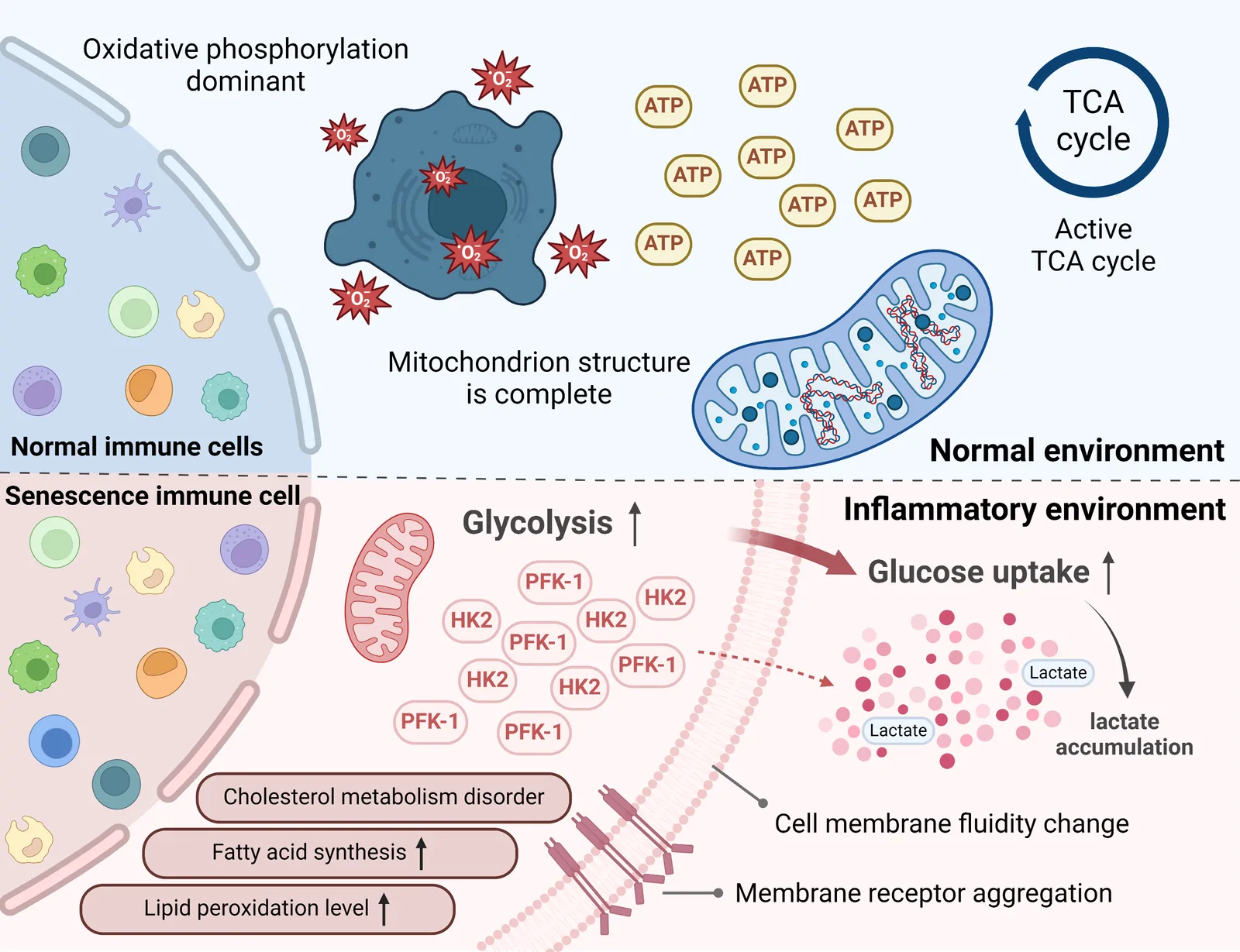
**Chronic inflammation-driven abnormalities in immune cell metabolism and their synergistic effects with SASP.** Under chronic inflammation, many abnormalities in immune cell metabolism occur. On the one hand, hyperglycolysis is obvious, and tumor cells take up glucose through GLUT1, which leads to accumulation of lactate, activation of GPR81 signaling, and consequently suppression of T cell function. On the other hand, lipid metabolism is also disturbed, and SREBP1/FASN-mediated lipid peroxidation induces impaired TCR signaling and NLRP3 inflammasome activation. Not only that, metabolism and epistasis also interact and reprogram, for example, succinate inhibits KDM5A, α-KG deficiency inhibits TETase, and locks depletion-related gene expression.

The pro-tumorigenic SASP effect is amplified by chronic inflammation through metabolic reprogramming in immune cells, characterized by hyperglycolysis, lipid dysregulation, and metabolic-epigenetic crosstalk (Fig. 4) [67]. Chronic inflammation induces immunosenescence via a glycolytic shift in CD8⁺ T cells and macrophages, marked by upregulated glycolytic enzymes and mitochondrial dysfunction [68]. Tumor cells exacerbate this process through GLUT1-mediated glucose competition, creating a lactate-rich microenvironment. Lactate not only suppresses T cell activity but also enhances IL-6 production via histone acetylation and stabilizes HIF-1α to upregulate PD-L1, linking metabolic rewiring to immune evasion and therapy resistance [69-71].

Lipid metabolic disorders further drive immunosenescence through cholesterol accumulation and fatty acid synthesis. Inflammation-triggered lipid peroxidation disrupts T cell membrane integrity and TCR signaling, while dysregulated cholesterol activates NLRP3 inflammasomes and IL-1β secretion, fostering pro-metastatic niches [72-75]. Clinical observational studies in colorectal cancer patients revealed a significant correlation between elevated serum cholesterol levels and increased tumor-infiltrating Treg proportions, suggesting a direct association between cholesterol metabolism disorders and the immunosuppressive microenvironment [76].

Metabolic-epigenetic interactions form a core axis of immunosenescence. Succinate accumulation inhibits histone demethylases to activate exhaustion-related genes, while α-ketoglutarate deficiency induces PD-1 promoter hypermethylation, locking T cells in an exhausted state [77-79]. The dynamic coupling between metabolic reprogramming and epigenetic modifications is stage specific, where early intervention can partially reverse senescence features, whereas late-stage modifications become irreversible [80]. Targeting glycolysis, lipid metabolism, and epigenetic modification networks may reverse immunosenescence. However, inhibiting glycolysis to activate metabolic compensatory mechanisms such as fatty acid oxidation remains a major challenge.

### 2.4. The microbiome

**Figure 5.**
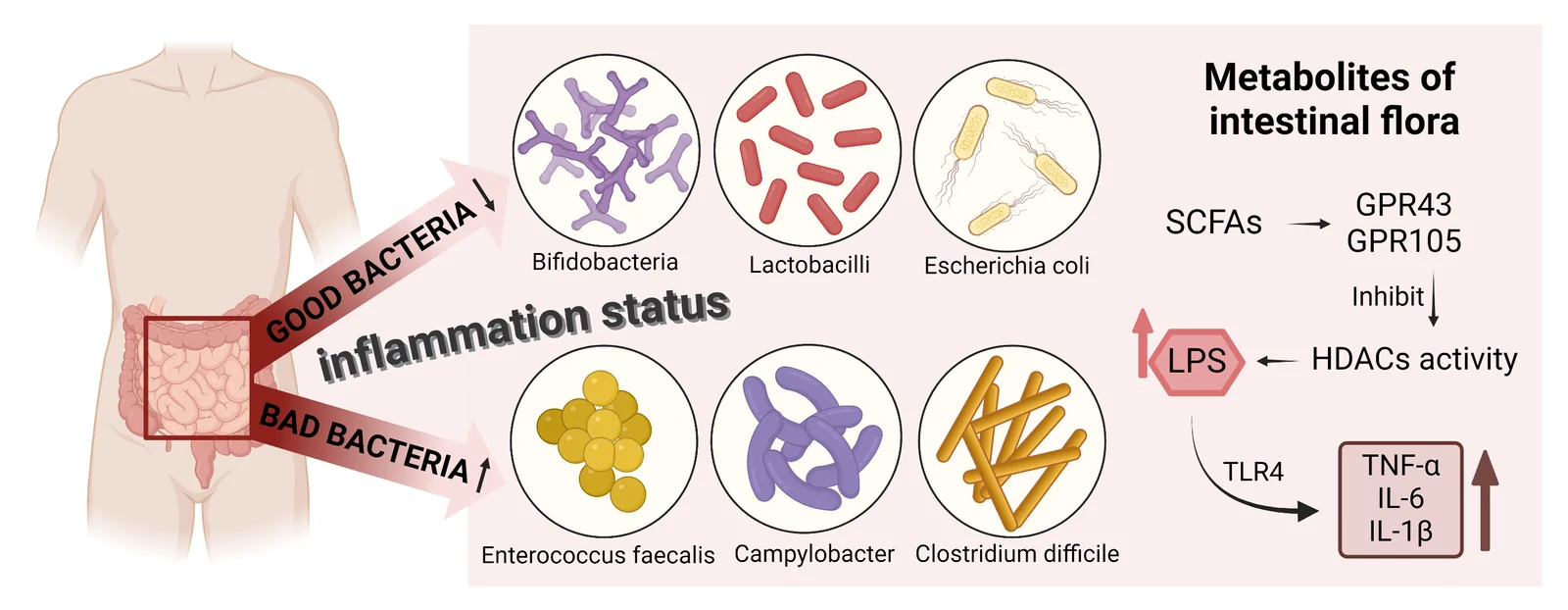
**Dysbiosis of intestinal flora affects tumor immunosenescence through metabolites and immune signaling pathways.** When the diversity of the flora is reduced, a decrease in SCFAs-producing bacteria and proliferation of pro-inflammatory bacteria disrupts the intestinal barrier, and LPS induces systemic inflammation through the TLR4/NF-κB pathway. In contrast, SCFAs (e.g., butyric acid) regulate Treg differentiation and CD8⁺ T cell function through HDAC inhibition and GPR signaling. In addition, harmful metabolites, like kynurenine and phenols, activate the AhR/TLR4 pathway and drive MDSCs expansion and T cell senescence.

The gut microbiome, through its metabolites and immunomodulatory effects, forms a dynamic network of interactions with the host immune system, profoundly influencing the inflammatory response and immunosenescence process. Disturbed metabolite profiles and gut barrier disruption due to dysbiosis are important drivers of tumor immune escape (Fig. 5).

In chronic inflammatory states, the diversity of the intestinal microbiome is significantly reduced, as evidenced by a decrease in the abundance of short-chain fatty acid-producing bacteria and an over-proliferation of pro-inflammatory bacteria, a phenomenon known as dysbiosis. Dysbiosis weakens the intestinal barrier function by disrupting the tight junction protein ZO-1, leading to bacterial translocation and activation of the TLR4/NF-κB signaling pathway, which induces a systemic storm of inflammatory factors [81]. Deficiency of intestinal flora metabolites, such as the short-chain fatty acid butyric acid, can exacerbate intestinal barrier disruption by inhibiting HDAC activity, leading to elevated levels of lipopolysaccharide (LPS) in the circulatory system, which can activate the Toll-like receptor 4 (TLR4) signaling pathway in immune cells, inducing the massive secretion of inflammatory factors such as TNF-α, IL-6, and IL-1β that further exacerbate the inflammatory response and the process of immunosenescence [82]. Clinical cohort studies have shown that the abundance of butyric acid-producing bacteria in the feces of colorectal cancer patients is negatively correlated with serum IL-6 levels, while oral administration of sodium butyrate restores intestinal barrier function and reduces circulating levels of inflammatory factors, suggesting a central role for bacterial metabolites in the regulation of SASP [83].

Gut microbes produce a variety of metabolites, such as short-chain fatty acids (SCFAs), bile acids (BAs), and tryptophan metabolites, which bidirectionally regulate immunosenescence through epigenetic reprogramming and immune signaling pathway modulation [84]. SCFAs such as butyric acid promote Treg differentiation by upregulating Foxp3 acetylation through inhibition of HDAC3, while activation of GPR43 enhances mitochondrial respiration and IFN-γ secretion in CD8⁺ T cells [85, 86], while propionic acid inhibits macrophage NLRP3 inflammasome activation through mTORC1 signaling [87]. Deoxycholic acid (DCA) inhibits IL-23 secretion from intestinal epithelial cells via FXR receptors and attenuates Th17 cell-mediated intestinal inflammation, whereas taurine-bound BAs promote IL-10 secretion from dendritic cells via TGR5 signaling to induce immune tolerance [88, 89]. Tryptophan metabolites such as indole-3-propionic acid induce PD-1 expression in CD8⁺ T cells through AhR signaling, whereas kynurenine promotes MDSCs expansion and inhibits NK cytotoxicity through activation of GPR35 [90-93]. In contrast, harmful bacterial metabolites, such as phenols and p-cresol, drive macrophage polarization toward pro-inflammatory phenotypes and accelerate T cell senescence through activation of the TLR4/MyD88 pathway and induction of mitochondrial dysfunction [94].

## 3. Inflammation-immunosenescence axis reshapes the TME

**Figure 6.**
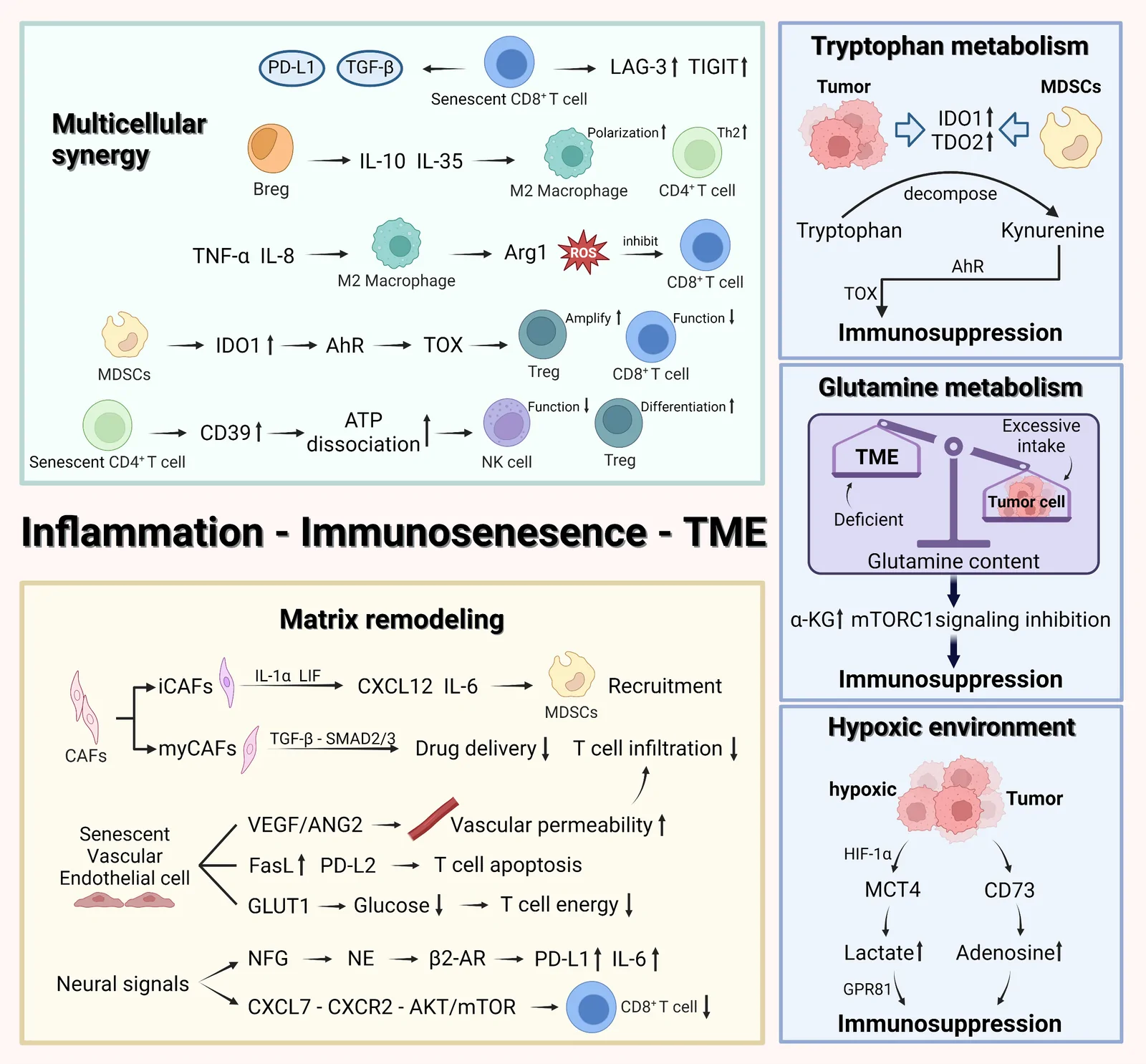
**Dynamic remodeling process of the tumor microenvironment driven by chronic inflammation and immunosenescence.** Immunosuppression hierarchical construction is an important aspect, with senescent T cells secreting PD-L1+ exosomes and MDSCs mediating tryptophan metabolism inhibition via IDO1. Meanwhile, stromal cells undergo pro-tumorigenic transformation, iCAFs secrete CXCL12 to maintain CSC stemness, and myCAFs form a dense stromal barrier. In addition, metabolic microenvironmental imbalance was prominent, with hypoxia/HIF-1α upregulating CD73 and glutamine deprivation inhibiting T cell proliferation. Moreover, spatio-temporal heterogeneity regulation is involved, with invasive frontier EMT⁺ tumor cells co-localizing with depleted T cells.

Chronic inflammation and immunosenescence systematically remodel the TME through a multidimensional interaction network, forming a complex pathological ecosystem characterized by immunosuppression, stromal abnormalities, and metabolic imbalance (Fig. 6). This remodeling not only promotes immune escape from the tumor, but also provides adaptive support for tumor progression by dynamically regulating intercellular interactions and signaling networks.

### 3.1 Hierarchical construction of immunosuppressive microenvironments

The inflammation-immunosenescence axis acts through cellular autoregulation to construct a tumor immunosuppressive ecology. Senescent immune-senescence cells play an active regulatory role in this process. Senescent CD8⁺ T cells secrete PD-L1^+^ exosomes at a significantly increased frequency compared with younger cells and directly inhibit the proliferation of neighboring T cells through the TGF-β/SMAD3 signaling axis [95, 96]. The scRNA-seq showed that senescent CD8⁺ T cells highly expressed the immune checkpoint molecules LAG-3 and TIGIT on their surface, forming a “checkpoint cluster” phenotype, which further weakened the anti-tumor response [97]. In addition, senescent regulatory B cells (Breg) drive M2-type TAM polarization through secretion of IL-10 and IL-35 and induce CD4⁺ T cells to shift toward a Th2 phenotype through inhibition of STAT1 phosphorylation [98, 99]. Further studies have shown that senescent Breg overexpress PD-L1 and form immunosuppressive synaptic structures [100].

Chronic inflammation also drives the differentiation of myeloid cells toward immunosuppressive subtypes that further construct the myeloid barrier. TNF-α and IL-8 are involved in the formation of an immunosuppressive microenvironment by regulating the polarization state of tumor-associated neutrophils (TANs) [101]. In the TME, TNF-α induces conversion of TANs to the N2 phenotype through activation of the NF-κB pathway, synergistically with IL-8, and inhibits CD8⁺ T cell function through Arg1-mediated metabolic inhibition and ROS-induced dysfunction [102, 103]. MDSCs upregulate IDO1 expression through STAT3-dependent metabolic reprogramming, catalyze the catabolism of tryptophan to kynurenine, and activate the AhR/TOX signaling axis to induce Treg amplification [104].

Cascading activation of a novel immune checkpoint network breaks through the limitations of traditional PD-1/CTLA-4 targeted therapy. V-domain immunoglobulin suppressor of T cell activation (VISTA) has been identified as a negative immune checkpoint molecule [105]. Structural analysis by cryo-electron microscopy revealed that VISTA indirectly inhibits TCR signaling through its IgV structural domain by forming a specific binding to the prostate stem cell antigen PSCA [106, 107]. In addition, senescent CD4⁺ T cells form a cascading inhibitory network through the CD39/CD73-adenosine axis. CD39 catalyzes the hydrolysis of ATP to generate AMP, which is further converted by CD73 to adenosine; adenosine inhibits NK cell killing function and promotes Treg differentiation via the A2AR-cAMP-PKA signaling axis [108, 109]. In addition, adenosine induces macrophage differentiation toward the PD-L1^hi^ phenotype, while cGAMP released by tumor cells enhances IFN-β secretion via the STING-IRF3 pathway, creating a self-sustaining immunosuppressive microenvironment [110, 111].

### 3.2 Tumor-promoting transformation and functional heterogeneity of stromal cells

Stromal cells undergo functional remodeling driven by the inflammation-senescence axis and become accomplices of pro-tumorigenic processes in the TME. Single-cell transcriptome analysis revealed the existence of functionally heterogeneous subpopulations of CAFs, including inflammation-associated subpopulations (iCAFs) and myofibroblast-like subpopulations (myCAFs). Driven by the IL-1α/LIF/IL-6 triple signaling, iCAFs recruit MDSCs and maintain CSC stemness by secreting CXCL12 and IL-8 to form a spatial concentration gradient[112]. myCAFs, on the other hand, secrete LOXL2-crosslinked collagen via TGF-β-induced YAP/TAZ nuclear translocation, forming a dense matrix barrier that impedes the migration rate of CD8⁺ T cells [44]. Studies have shown that in breast cancer, iCAFs are predominantly localized in the tumor core, whereas myCAFs are enriched in the invasive front. This difference in spatial distribution determines the intensity of local immunosuppression and significantly correlates with patient prognosis [113].

The immunosenescence of tumor vascular endothelial cells builds an immunosuppressive microenvironment through multiple mechanisms. Dysregulation of VEGF/ANG2 signaling leads to vascular leakage, and fibrinogen deposition creates a physical barrier that impedes effector T cell infiltration [114]. Endothelial cells induce apoptosis and depletion of infiltrating T cells through high expression of FasL and PD-L2, while inhibiting DC maturation through secretion of semaphorin 4D [115]. In addition, metabolic hijacking is one of the important mechanisms. Endothelial cells in the tumor core region upregulate excessive glucose uptake via mTORC1-dependent GLUT1 upregulation, forcing T cells to turn into fatty acid oxidation and lose effector function [116].

Furthermore, reprogramming of the neuro-immune axis provides a new regulatory dimension for tumor immune escape. Chronic inflammation promotes sympathetic nerve fiber invasion into the TME by inducing nerve growth factor (NGF) release. Norepinephrine (NE) released from nerve endings triggers the cAMP-PKA-CREB signaling cascade response by activating tumor cell β2-adrenergic receptors (β2AR), leading to the upregulation of PD-L1 expression and promotion of IL-6 secretion, forming a neuro-immunosuppression axis [117, 118]. In pancreatic cancer, spatial multi-omics showed a positive correlation between nerve density and the expression of CD8⁺ T cell exhaustion markers (TIM-3⁺LAG-3⁺) [119]. Mechanistically, neuronal secretion of CXCL12 activates the AKT/mTOR pathway via the CXCR4 receptor, induces elevated TOX expression, and inhibits mitochondrial function [120]. In a mouse model of pancreatic cancer, CXCR4 antagonist AMD3100 combined with anti-PD-1 treatment increased the response rate from 22% to 65% and significantly prolonged median survival [121, 122].

### 3.3 Spatio-temporal correlation between immune-senescence and tumor immune escape

Immunosenescence confers tumor immune escape plasticity through spatio-temporal dynamic regulation. This process is a dynamic coupling of “Darwinian” evolutionary pressures and epi-metabolic remodeling in the tumor ecosystem. One of the key mechanisms is the all-encompassing suppression of antigen presentation. Tumor cells directly silence the B2M gene through EZH2-mediated H3K27me3 modification, resulting in decreased MHC-I expression [123]. Significantly, this episodic silencing is spatially heterogeneous. Invasive frontline tumor cells maintain partial MHC-I expression through H3K4me3 modification to escape T cell immune surveillance while avoiding NK cell activation to balance immune escape with with NK cell activation risks. In addition, downregulation of the expression of the immunoproteasome subunits PSMB8/9 can lead to a reduction in the diversity of tumor antigenic peptides, which can impair the specific response of CD8⁺ T cells [124].

Cascading activation of immune checkpoint networks also plays a role in immune escape. The inflammation-immunosenescence axis activates non-classical immune checkpoint pathways, such as the TIM-3/Galectin-9 axis. Senescent T cells upregulate the TIM-3/Galectin-9 axis through epigenetic memory, driving YAP nuclear translocation and inducing TOX expression through inhibition of LATS1/2 kinase activity, thereby creating “epigenetic lock-in” of the depletion phenotype [125, 126]. In contrast, phosphorylation modification of SIRPα on the macrophage surface remodels the CD47-binding interface and activates IL-10^hi^ Treg amplification while inhibiting phagocytosis, revealing a longitudinal network of checkpoint signaling from membrane receptors to epistatic regulation [127]. In addition, tumor cells secrete exosomes carrying PD-L1 and tumor antigens, which also induce T cell apoptosis and deplete antigen-specific T cell clones [128].

These spatio-temporally heterogeneous regulatory mechanisms collectively promote immune selection pressure, favoring the survival of tumor clones with reduced immunogenicity. Following T cell-mediated elimination of neoantigen-high tumor populations, residual cells acquire adaptive traits such as downregulation of MHC-I or activation of the epithelial-mesenchymal transition (EMT), thereby evading immune surveillance and sustaining tumor progression under immunological stress [129]. Meanwhile, ZEB1-mediated EMT confers stem cell properties to tumor cells and enhances their resistance to T cell killing by downregulating MHC-I expression [130]. Spatial transcriptomics studies have shown that EMT⁺ tumor cells at the invasion front are significantly associated with co-localization of CD8⁺ T cell exhaustion markers, such as LAG-3⁺, PD-1⁺, suggesting a central role for phenotypic plasticity in spatio-temporal escape [97, 131, 132].

### 3.4 Reprogramming of the metabolic micro-environment and immunosuppression

Metabolic reprogramming forms a positive feedback loop with the inflammation-aging axis that synergistically drives immunosuppression through multidimensional mechanisms. Dysregulated tryptophan metabolism is a key component of this, with tumor cells and MDSCs highly expressing IDO1/TDO2, which catalyzes the catabolism of tryptophan to kynurenine, which induces Treg differentiation and upregulates TOX expression through activation of the AhR signaling pathway and promotes the differentiation of CD8⁺ T cells toward a depleted phenotype [133]. Phase I/II clinical trials reported that combining the IDO1 inhibitor Epacadostat with anti-PD-1 therapy partially reversed Treg-mediated immunosuppression in melanoma patients [134]. In addition, imbalance of glutamine metabolism further exacerbates immunosuppression. Excessive glutamine uptake by tumor cells via ASCT2/SLC1A5 leads to microenvironmental glutamine deprivation, inhibits mTORC1 signaling and hinders T cell proliferation and effector functions [104]. Meanwhile, restricted glutamine metabolism also leads to decreased α-KG levels, which inhibits TET enzyme activity, resulting in hypermethylation of the IFN-γ gene promoter, which further impairs the anti-tumor activity of T cells [135]. The hypoxic microenvironment then remodels metabolism and immune response through HIF-1α signaling, upregulates CD73 expression to promote adenosine production, and inhibits NK cell and CD8⁺ T cell function through the A2AR-cAMP-PKA axis [136], whereas HIF-1α-induced MCT4 expression accelerates lactate efflux and synergizes with GPR81 signaling to inhibit T cell activity [137, 138]. This hypoxic environment not only limits the activity of immune cells, but also provides a survival advantage for tumor cells, further promoting immunosuppression [139]. Notably, metabolic interventions may trigger compensatory pathway activation. For example, inhibition of IDO1 may upregulate TDO2 expression, and blocking glutamine uptake may enhance lipid metabolism dependence in tumor cells [140]. Therefore, metabolic modulation needs to be combined with multi-omics analysis to systematically assess compensatory risk.

## 4. Anti-tumor strategies targeting the inflammation-immunosenescence axis

**Figure 7.**
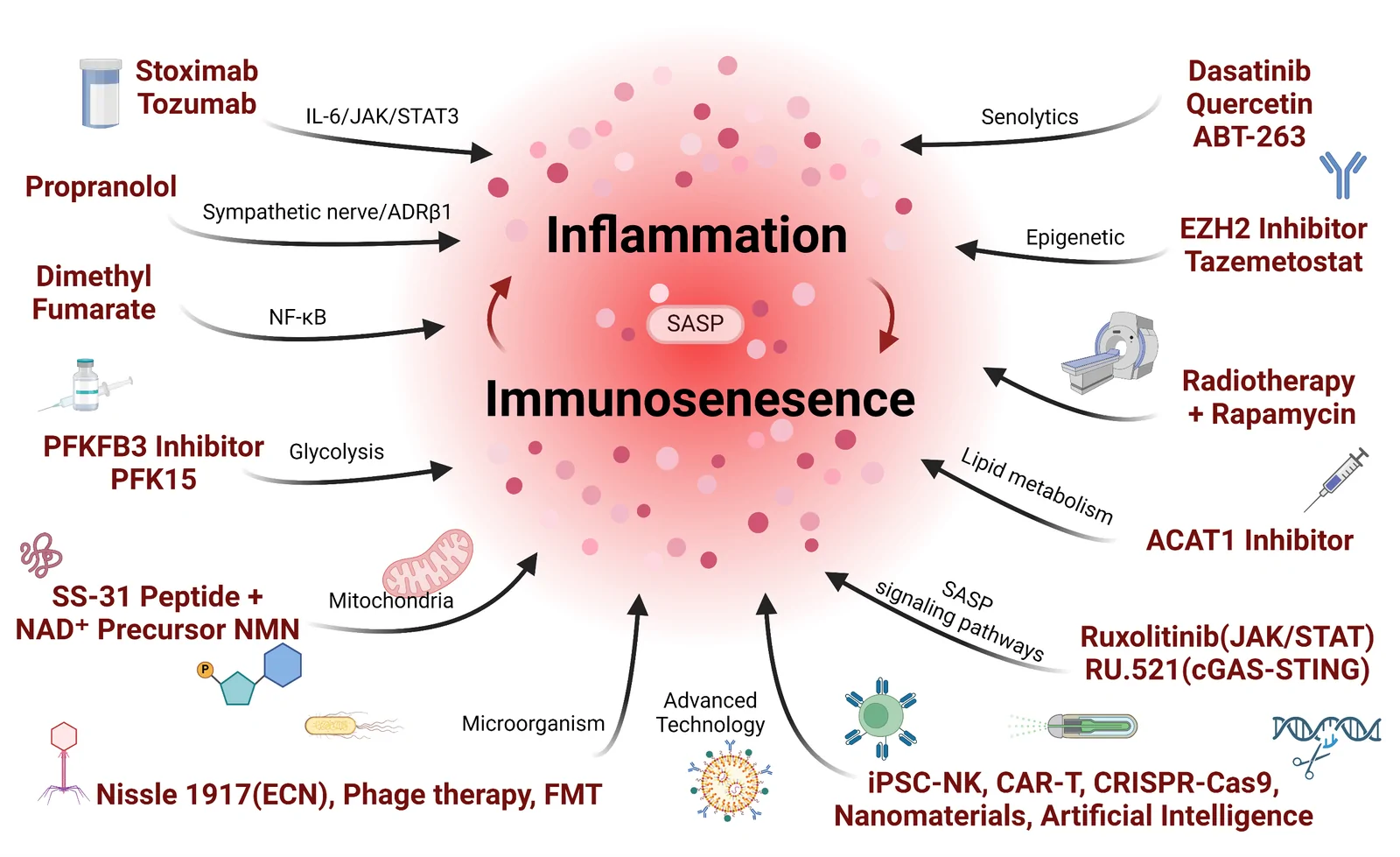
**Multidimensional intervention strategies for chronic inflammation-driven immunosenescence.** For source-targeted intervention, there are anti-IL-6 antibodies, ROS scavengers, and colony transplantation. For SASP network blockade, Senolytics drugs, JAK/STAT inhibitors are available. In terms of combination therapy optimization, combination of epigenetic drugs and immune checkpoint inhibitors and radiotherapy with SASP modulation are common approaches. In addition, there are technological innovations such as CRISPR-edited CAR-T and nano-delivery systems.

Chronic inflammation-driven immunosenescence is one of the core mechanisms of tumor immune escape, and its dynamic regulatory network involves multidimensional pathological processes such as inflammatory factor storm, metabolic reprogramming, SASP activation, and microbiome interactions. Intervention strategies to address this complex network need to take into account spatial and temporal heterogeneity and safety balance, modulate inflammatory signals at the source, block the SASP cascade effect, optimize the combination-therapy modalities, and achieve precise intervention with the help of technological innovation (Fig. 7).

### 4.1 Source intervention: Anti-inflammatory and metabolic regulation

Persistent stimulation of chronic inflammation is the initiator of immunosenescence, and targeting key inflammatory mediators and metabolic abnormalities is the cornerstone of reversing the aging process. Core drivers of chronic inflammation are important targets for intervention. IL-6-induced T cell exhaustion through the JAK/STAT3 pathway has demonstrated potential in clinical trials, and stuximab targeting IL-6, as well as tocilizumab targeting IL-6R, have emerged as promising immunotherapeutic agents for tumor treatment, either as stand-alone therapies or in combination with conventional chemotherapy [141, 142]. Studies have shown that tocilizumab significantly reduces circulating IL-6 levels and enhances the efficacy of PD-1 antibodies in patients with metastatic colorectal cancer [143].

However, the inhibition of pro-inflammatory factors alone may weaken the strength of the immune response and need to be combined with metabolic modulation to enhance efficacy. For example, mitochondrial dysfunction is one of the central features of immunosenescence, and ROS scavengers can reduce oxidative stress, enhance mitochondrial integrity, and shift macrophages from a pro-inflammatory to an anti-inflammatory state [144]. The mitochondria-targeting peptide SS-31 reduces ROS accumulation by stabilizing the mitochondrial membrane potential, and in combination with the NAD⁺ precursor NMN significantly restores the oxidative phosphorylation capacity of T cells and reverses the depletion phenotype [145, 146]. In addition, the NRF2-agonist dimethyl fumarate slows down the process of immunosenescence by activating antioxidant response elements (AREs), inhibiting NF-κB-driven inflammatory signaling, and restoring mitochondrial function in T cells [147]. Chronic inflammation induces sympathetic hyperactivation directly induces T cell exhaustion through activation of the β1-adrenergic receptor (ADRβ1), and the β-blocker propranolol reduces the formation of depleted T-cells and enhances tumor infiltration of CD8⁺ T-cells after blocking ADRβ1 [148]. Such strategies not only target inflammation itself, but also achieve multidimensional regulation through metabolic-neuro-immune interaction networks. Inhibition of the glycolysis rate-limiting enzyme PFKFB3 enhances persistence by reducing glycolysis and forcing T cells to rely more on oxidative phosphorylation [149]. In a study, a PFKFB3 inhibitor (PFK15) in combination with a PD-1 monoclonal antibody inhibited tumor cell growth by enhancing the activity of CD8⁺ T cells. This suggests that PFKFB3 inhibitors may enhance the effects of immunotherapy by modulating metabolic pathways in the TME [150]. Lipid metabolism regulation is equally important, and inhibition of the cholesterol esterase ACAT1 is a key metabolic checkpoint in the regulation of T cell anti-tumor immunity. Inhibition of ACAT1 enhances the tumor-killing capacity of CD8⁺ T cells, mainly by increasing free cholesterol levels in the cell membrane and improving the efficiency of TCR signaling [151].

The potential for precision intervention of gut flora as a key regulator of the inflammation-aging axis is emerging. The synthetic biology-modified butyrate-synthesizing bacterium Escherichia coli Nissle 1917 (ECN) is able to exert its probiotic effects by secreting antimicrobials, modulating intestinal epithelial barrier function, and promoting the secretion of anti-inflammatory cytokines, such as IL-10, by restoring the intestinal barrier function and reducing the level of circulating LPS to reshape the intestinal immune homeostasis [152, 153]. In the clinical sample study and mouse model of colorectal cancer, synthetic biology-engineered butyrate-producing bacteria reduced tumor burden and synergized with anti-PD-1 therapy [154]. In addition, phage therapy attenuates inflammation by selectively removing pro-inflammatory flora, such as Clostridium perfringens [155]. It has been suggested that the discovery of certain bacterial antigens mimicking tumor neoantigens may enable the isolation and in vitro expansion of T cells targeting cross-reactive, microbe-derived peptides for use in overt T cell therapy [156]. In addition, Fecal Microbial Transplantation (FMT) was found to be able to enhance the efficacy of immune checkpoint inhibitor (ICI) by restoring the diversity of intestinal flora and reducing circulating LPS levels, which in turn improves the systemic inflammatory state [157, 158]. These studies suggest that microbiome modulation is not only an anti-inflammatory tool, but also a hub for remodeling systemic immune homeostasis. However, the tissue specificity of the metabolic effects of the bacterial population and differences in host genetic backgrounds lead to heterogeneity in therapeutic response, and a combination of macro-genomics and metabolomics is needed to guide precise dosing.

### 4.2 SASP network blocking

Accumulation of senescent cells drives the immunosuppressive microenvironment through SASP factors, and senolytics drugs may exhibit therapeutic potential by selectively removing senescent cells (Table 2). Dasatinib in combination with quercetin inhibits pro-survival networks such as BCL-2/BCL-XL in senescent cells and induces apoptosis in tumor cells [159-161]. This combination, combined with anti-PD-1 therapy, resulted in a significant reduction in both tumor size and MDSC infiltration in melanoma mice [162]. Furthermore, the BCL-2 inhibitor ABT-263 (Navitoclax) selectively removed chemotherapy-induced senescent cells in a breast cancer model, thereby inhibiting tumor progression. In addition, ABT-263 promotes apoptosis of senescent cells and improves tumor response after chemotherapy by targeting anti-apoptotic proteins such as BCL-XL and BCL-2 [163]. However, the lack of tissue specificity of senolytics may lead to normal cell damage, limiting their clinical application.

The hierarchical blockade of the SASP signaling pathway is another important direction. The JAK/STAT and p38 MAPK pathways are co-regulators of SASP. The JAK inhibitor Ruxolitinib inhibits IL-6/STAT3 signaling and reduces the secretion of SASP factors, which has been demonstrated in several disease models including myelofibrosis and atopic dermatitis, where ruxolitinib significantly reduces secretion of inflammatory factors through inhibition of the JAK/STAT pathway [164]. Targeting the cGAS-STING pathway blocks mtDNA-driven SASP amplification. The small molecule inhibitor RU.521 attenuates the pro-inflammatory effects of SASP by inhibiting cGAS activity and reducing type I interferon secretion [165, 166].

### 4.3 Combination therapy optimization

Single-targeted strategies often limit efficacy due to compensatory mechanisms or heterogeneity, making combination therapy a necessity. The combination of epigenetic drugs with immune checkpoint inhibitors shows synergistic potential. The EZH2 inhibitor Tazemetostat improved the efficacy of CAR-T cells and TCR-T cells against a wide range of hematological tumors and solid tumors through epigenetic reprogramming [167]. Radiotherapy is used synergistically with SASP modulation strategies. Rapamycin, an mTOR inhibitor, has been shown to reduce SASP secretion by inhibiting the mTOR signaling pathway. Local radiotherapy is able to induce tumor cells to enter a senescent state, and the use of rapamycin is able to inhibit SASP secretion from senescent fibroblasts, thereby diminishing its promotional effect on tumor growth [168].

**Table 2:** Therapeutic strategies entering preclinical studies.

Therapeutic strategy	Drugs/Methods	Target	Research Models	NCT Number
**Blocking the tumor-promoting effect of SASP signaling pathway**	ABT-263 (Navitoclax)	senescent cell	Breast cancer	NCT00406809
	Ruxolitinib	IL-6/STAT3	Myelofibrosis model	NCT00952289
	Rapamycin	mTOR	Multiple hematologic and solid tumors	NCT01106833
				NCT01488253
				NCT05144698
	siltuximab	IL-6	Metastatic pancreatic adenocarcinoma	NCT04191421
	TTI-101	IL-6/JAK/STAT	Malignant solid tumors	NCT03195699
	tocilizumab	IL-6	Complications of advanced cancer	NCT06016179
	Curcumin	IL-6, -8 and -10	Adenocarcinoma and advanced pancreatic cancer	NCT00094445
**Epigenetic**	Tazemetostat	EZH2	Multiple hematologic and solid tumors	NCT03525795
				NCT04241835
				NCT01897571
**Cell therapy**	CAR-T cell	IL-10/CAR-T cell	Multiple hematologic and solid tumors	NCT02872116
				NCT05008783
				NCT04950322
	iPSC-NK	NK cell	Hematologic tumor models (AML, multiple myeloma)	NCT04023071

However, the toxicity overlay and patient heterogeneity of combination therapy remain a clinical-translational bottleneck. In a phase III clinical trial, Epacadostat in combination with the anti-PD-1 agent Keytruda did not show a significant progression-free survival benefit, which may be related to the dosing regimen and experimental design, suggesting the need for the development of biomarker-guided precision dosing [169]. While the strategy of PD-1 antibodies combined with IDO1 inhibitors enhances efficacy, the sensitivity of different tumor types to the combination regimen varies significantly. For example, IDO1 inhibitors have an unclear effect in melanoma but show more significant anti-tumor potential in colorectal cancer [170]. Furthermore, although inhibition of IDO1 can temporarily reverse immunosuppression, tumor cells may rebuild immune escape by up-regulating alternative pathways, such as TDO2 or lipid metabolism [171-173]. Collectively, it is necessary to develop a multi-targeted combination strategy or combine metabolic reprogramming with immune checkpoint inhibitors, but the toxicity superposition and patient tolerance of such schemes still need to be further evaluated.

### 4.4 Innovative technology applications

In the field of novel technology applications, breakthroughs in gene editing technology have opened up innovative paths for immunotherapy. By modifying the genetic properties of immune cells, researchers have successfully endowed them with anti-immunosenescence ability- iPSC-differentiated NK cells (iPSC-NK) have high telomerase activity, which makes them resistant to replicative senescence and exhibit longer-lasting activity in vivo, and they have demonstrated strong killing ability and durability in hematological tumors demonstrating potent killing ability and durability [174, 175]. In addition, it was shown that by knocking down the NKG2A gene in NK cells, their anti-tumor and anti-viral abilities could be significantly enhanced. Knockdown of the NKG2A gene was able to deregulate the immunosuppression of NK cells by cancer cells through the NKG2A-HLA-E pathway, thereby increasing the cytotoxicity of NK cells [176]. CAR-T cells overexpressing IL-10 inhibit local inflammation and prolong survival in vivo. In both syngeneic and xenograft models of various cancer types, including colorectal, breast, melanoma, and pancreatic cancers, IL-10 overexpressing CAR-T cells not only achieved tumor regression, but also induced memory T-cell responses, which provided long-term anti-tumor protection for the organism [177]. CAR-T cells overexpressing superoxide dismutase 2 (SOD2) exhibited significant antioxidant damage properties, and their survival time in mitochondrial ROS-enriched solid TME was prolonged by up to threefold, which provided a new idea for solid tumor therapy [178].

However, CAR-T cell therapy may induce cytokine release syndrome (CRS). Therefore, it is crucial to develop technologies with higher anti-tumor effects. Genome editing technologies, particularly clustered regularly interspaced short palindromic repeats (CRISPR)-Cas9, which precisely regulate genes involved in immunosenescence, are being used to create CAR-T cells that are highly resistant to immune cell suppressor molecules. It has been shown that CRISPR-Cas9-based in vivo epigenetic editing to knock down the SUV39H1 gene in T cells can enhance the anti-tumor effect and in vivo persistence of CAR-T cells, and effectively reverse T cell exhaustion [179]. The transcription factor FOXO1 has been shown to promote memory formation and prevent depletion of CAR-T cells, thereby enhancing their anti-tumor activity, and overexpression of FOXO1 increases the anti-tumor activity of human CAR-T cells [180, 181]. Another study identified multiple epigenetic factors associated with T cell exhaustion through a genome-wide CRISPR-Cas9 screen. For example, perturbation of INO80 and BAF chromatin remodeling complexes improves T cell persistence in tumors, and deletion of BAF complex members, such as Arid1a, downregulates depletion-associated genes and enhances anti-tumor immunity [182].

In addition, big-data-driven by the advent of the artificial intelligence era and the widespread use of histological technologies has broadened and deepened the understanding of immunosenescence, and the *Immunosenescence Inventory* (https://ngdc.cncb.ac.cn/iaa/home), a multi-omics database for immunosenescence research, is now online and provides a comprehensive resource for researchers [183]. The exquisite design of the nano-delivery system provides technical support for targeting and regulating the TME. Using the novel delivery platform, it was able to precisely target TAMs, promote the conversion of M2-type TAMs to M1-type in TME through downregulation of CCL5, and then enhance the infiltration of CD8+ T cells, remodel the tumor immune microenvironment, and effectively inhibit the recurrence and metastasis of pancreatic ductal adenocarcinoma (PDAC) after surgery [184]. 3D printing technology has also been used to construct bionic scaffolds with immunomodulatory functions. These scaffolds can remodel the immune microenvironment by locally delivering drugs or bioactive molecules, a technique that provides a spatially precise regulatory scheme for reprogramming the local immune microenvironment [185]. These technological breakthroughs not only expand the anti-tumor arsenal, but also bring light to overcoming immunosenescence-associated therapeutic resistance through multidimensional synergistic strategies.

## 5. Challenges and future directions

The clinical translation of chronic inflammation-driven tumor immunosenescence mechanisms faces multiple challenges, including patient heterogeneity, treatment safety, and stability of therapeutic efficacy. These issues are particularly pronounced when translating these therapeutic strategies to elderly patients, who face profound challenges rooted in immunosenescence. Aging individuals often exhibit baseline immune dysfunction due to comorbidities such as thymic atrophy, chronic inflammation, and cardiovascular diseases, leading to impaired immune cell functionality and reduced treatment tolerance [186, 187]. For instance, while senolytic agents effectively clear senescent cells, preclinical studies in aged mouse models have revealed adverse effects such as diminished tissue repair capacity and delayed wound healing [188]. Similarly, the efficacy of ICIs in elderly patients presents complexities. Compared to younger cohorts, elderly patients exhibit attenuated cytokine dynamics in peripheral blood during ICI therapy, suggesting a blunted immune response [189].

Age-related alterations in gut microbiota composition, often accompanied by compromised intestinal barrier integrity, exacerbate systemic inflammation and complicate microbiota-based interventions in the elderly [190]. Common comorbidities such as cardiovascular diseases may amplify drug toxicity risks, yet current studies rarely stratify outcomes by age [191]. Furthermore, the spatio-temporal heterogeneity of the TME renders immunosenescence regulatory networks highly dynamic and context-dependent. For instance, distinct regions within the same tumor, such as the tumor core, invasive front, and metastatic niches, exhibit significant differences in inflammatory signaling gradients, immune cell senescence states, and SASP factor profiles. While existing technologies like single-cell multi-omics can resolve cellular heterogeneity, they remain limited in capturing spatio-temporal dynamics. The context-dependent nature of SASP factors implies that single-target strategies may fail due to tumor type or microenvironmental variations. Off-target toxicity also warrants critical attention. Tocilizumab, an anti-inflammatory agent modulating immune cells, may transiently suppress neutrophil function during initial treatment phases [192].

These findings collectively underscore the need for precision strategies tailored to the unique immunosenescence landscape of elderly patients. Despite the remarkable therapeutic potential of emerging technologies, their clinical translation necessitates cautious consideration of the inherent limitations and unresolved challenges. In the Phase III trial of Epacadostat combined with anti-PD-1 therapy (NCT02752074), unstratified patient cohorts and dynamic SASP heterogeneity contributed to the lack of significant progression-free survival improvement. The application of CRISPR-Cas9 systems in aging-related diseases faces challenges, including genomic instability caused by off-target effects, low tissue-specific delivery efficiency, and inefficacy in non-dividing cells [193]. CAR-T cell therapy in solid tumors is similarly constrained by immunosuppressive tumor microenvironments and physical barriers [194]. While nanoparticle delivery systems enhance targeting precision, they may trigger unintended immune activation or organ toxicity, as exemplified by liver accumulation of lipid nanoparticles observed in vivo [195].

Overcoming the current limitations in tumor immunosenescence research requires synergistic development of innovative therapies and interdisciplinary technological integration. First, technological innovation serves as the core driver to break through existing bottlenecks. For example, developing antibody-drug conjugates (ADCs) targeting senescence-associated surface markers can precisely modulate the local inflammatory microenvironment while minimizing systemic side effects. Alternatively, utilizing nanoparticle carriers may enhance drug enrichment efficiency in the tumor microenvironment (TME) and reduce off-target damage to healthy tissues, achieving TME-specific delivery [196, 197]. Integrating spatial transcriptomics with multiplex immunofluorescence enables the elucidation of spatio-temporal distribution patterns and interactions between tumor and immune cells within the TME [198]. Furthermore, optimizing organoid-immune cell co-culture systems allows simulation of the complex features of elderly patients’ TME, accelerating drug screening and mechanistic investigations [199, 200].

Finally, interdisciplinary collaboration is a must for the development of the field. Chronic inflammation-driven tumor immunosenescence is a highly dynamic and heterogeneous pathological process, and its mechanism analysis and clinical translation require multidisciplinary cross-collaboration. The cross-fertilization of immunology, metabolomics, microbiomics and engineering is giving rise to novel therapeutic paradigms. For example, CRISPR-Cas9-mediated in vivo gene editing technology is capable of precisely reversing epigenetic abnormalities associated with T cell exhaustion [201]. However, clinical translation of these emerging technologies demands rigorous evaluation of long-term safety, particularly in elderly populations. Future research must balance the reversal of immunosenescence with the maintenance of tissue homeostasis, with special consideration for age-related factors. Priority should be given to developing aging-specific preclinical models and incorporating assessments of therapeutic safety and efficacy stability into clinical trial designs to optimize outcomes and improve health in older individuals. Additionally, to achieve biomarker-driven clinical stratification, standardized evaluation protocols must be established to identify patients likely to benefit from specific therapies, emphasizing dynamic treatment adaptation. Only through the deep integration of mechanism exploration, technological innovation and clinical validation, focusing on the analysis of spatio-temporal heterogeneity, the development of personalized therapeutic strategies and the optimization of safety, then can we realize the paradigm shift from “generalized treatment” to “precise reversal of immunosenescence” and provide a new breakthrough for attacking tumor immunosenescence.

## Data Availability

All data that led to the conclusions in this manuscript have been referenced and all sources have been described.
